# Perturbation of Auxin Homeostasis by Overexpression of Wild-Type *IAA15* Results in Impaired Stem Cell Differentiation and Gravitropism in Roots

**DOI:** 10.1371/journal.pone.0058103

**Published:** 2013-03-05

**Authors:** Da-Wei Yan, Jing Wang, Ting-Ting Yuan, Li-Wei Hong, Xiang Gao, Ying-Tang Lu

**Affiliations:** State Key Laboratory of Hybrid Rice, College of Life Sciences, Wuhan University, Wuhan, Hubei, China; Iwate University, Japan

## Abstract

Aux/IAAs interact with auxin response factors (ARFs) to repress their transcriptional activity in the auxin signaling pathway. Previous studies have focused on gain-of-function mutations of domain II and little is known about whether the expression level of wild-type Aux/IAAs can modulate auxin homeostasis. Here we examined the perturbation of auxin homeostasis by ectopic expression of wild-type *IAA15*. Root gravitropism and stem cell differentiation were also analyzed. The transgenic lines were less sensitive to exogenous auxin and exhibited low-auxin phenotypes including failures in gravity response and defects in stem cell differentiation. Overexpression lines also showed an increase in auxin concentration and reduced polar auxin transport. These results demonstrate that an alteration in the expression of wild-type *IAA15* can disrupt auxin homeostasis.

## Introduction

As a growth-promoting hormone, auxin is vital for plant growth and development including root stem cell niche maintenance and gravity sensing. The root stem cell niche is composed of the mitotically less active quiescent center (QC) together with its neighboring stem cells and functions as a reservoir for the generation of all cells within the root [1], [2], [3]. In *Arabidopsis*, the maintenance of the root stem cell niche is orchestrated by auxin and several transcription factors [4]. Homeobox gene *WUSCHEL-RELATED HOMEOBOX5 (WOX5)* is specifically expressed in the QC and represses the differentiation of the columella stem cells (CSC) [5]. Another important regulator of the root stem cell niche belongs to the AP2-domain group of transcription factors *PLETHORA (PLT)*. The double mutant *plt1-4plt2-2* has reduced QC identity resulting in a higher proportion of differentiated columella cells [6], [7]. Genetic studies show that *PLTs* are epistatic to *WOX5* and auxin promotes the differentiation of CSC by repressing *WOX5* through *ARF10/ARF16* [4].

In response to gravity, roots grow downwards to absorb water and nutrients from the soil. According to the classic starch statolith hypothesis, the starch-filled amyloplasts in statocytes of the root tip function as statoliths [8], [9], [10], [11], [12], [13]. When placed in a horizontal position, the sedimentation of statoliths on the new bottom of statocytes, transmits a signal that influences physiological changes such as local pH [14], [15], Ca^2+^ concentration [16], [17] and auxin gradients [18], [19]. The following responses inhibit cell expansion in the lower side of the root tip, causing the root to bend towards gravity [18], [20].

It is well known that auxin functions as a positive regulator in gravity sensing [12], [21], [22], [23]. Asymmetrical application of auxin at one side of the root elongation zone alters tropism [24], [25], [26]. Several agravitropic mutants also indicate the involvement of the auxin signaling pathway [27], [28], [29]. For example, the double mutant of *ARF7* and *ARF19* displays abnormal gravitropic response [30]. In addition, the agravitropic behavior induced by the application of auxin transport inhibitor naphthylphthalamic acid (NPA) [31] highlights the importance of polar auxin transport (PAT) in response to gravity [32]. The impaired gravitropic responses in mutants of auxin influx AUXIN RESISTANT 1 (AUX1) [33], [34], [35] and PIN-FORMED (PIN) efflux facilitators PIN2 [36], [37], and PIN3 [20] further emphasize the requirement of PAT in root gravitropism.

It has been widely accepted that polar auxin transport (PAT) from IAA source to sink plays a vital role in establishing auxin gradients [21], [38], [39], [40]. However, recent reports argue that locally synthesized auxin also contributes in formation of auxin gradient [41], [42], [43], [44]. Additionally, IAA can be released from IAA-conjugates through hydrolytic cleavage, contributing to local auxin concentration [45], [46], [47]. Taken together, local auxin homeostasis depends on a combination of auxin biosynthesis, conjugation and PAT [47], [48]. The dynamic integration of auxin homeostasis and auxin signaling is required for plants to respond to various environmental changes or developmental processes [39], [49].

The auxin signaling pathway is well established in *Arabidopsis*. Auxin is perceived by auxin receptors TIR1/AFB1/AFB2/AFB3 [50], [51], which target the Aux/IAAs repressors for their degradation [52], [53]. Once Aux/IAAs are degraded, and Aux/IAA-mediated inhibition of ARFs is released, ARFs are free to activate downstream genes by binding to the auxin-responsive elements (AuxREs) [54] in the promoter regions of auxin response genes. The *Aux/IAAs* gene family has 29 members in *Arabidopsis*, with overlapping but distinct functions. Most Aux/IAAs have four conserved domains (I, II, III, and IV) [55], [56]. Domain I contributes to the repressive activity of Aux/IAAs. Domain II can be targeted by the ubiquitin-ligase SCF^TIR1^ for degradation and is thus vital for the regulation and stability of this protein [57]. Domain III and Domain IV are involved in homo- and heterointeraction with Aux/IAAs or ARFs [54].

To date, most functional studies of *Aux/IAAs* focus on the gain-of-function analysis of domain II. Mutations in the highly conserved amino acid sequence (VGWPPV) in domain II prevent Aux/IAAs from being targeted by SCF^TIR1^, and further influence the stability of these proteins. The mutagenized Aux/IAAs constantly bind to the downstream ARFs and silence their activities in various biological processes, resulting in diverse auxin-related phenotypes. For example, *axr3-1*, a gain-of-function mutation of *IAA17*, has defects in gravitropic response and lateral root formation [58]. It is interesting that repressed auxin signaling in *slr-1* results in an increase of auxin concentration in the root tip [59]. At the transcriptional level, overexpression of the domain II-less Aux/IAA proteins causes dramatic phenotypic changes [60], [61]. It was proposed that the relative long-lived properties of such non-canonical Aux/IAA proteins are responsible for auxin-related defects [62]. In contrast, overexpression of canonical wild type *Aux/IAAs* results in no obvious phenotype in some cases [63], [64]. However, there are also reports of phenotypic changes caused by overexpression of canonical wild type *Aux/IAAs* [65], [66]. Mechanisms underlying these phenotypic changes remain to be elucidated.

By ectopic expression of a canonical *Aux/IAA*, *IAA15*, we provide evidence that overexpression of wild type *Aux/IAAs* can modify auxin homeostasis. The overexpression lines were less sensitive to exogenous auxin and showed low-auxin phenotypes including reduced apical dominance and agravitropic response. *IAA15* overexpression also caused impaired stem cell differentiation and small meristem size, possibly by altering the expression of *WOX5* and *PLT1* respectively. Furthermore, overexpression lines showed an increase in auxin concentration and reduced polar auxin transport.

## Results

### Overexpression of Wild-type *IAA15* Resulted in Pleiotropic Phenotypes

**Figure 1:**
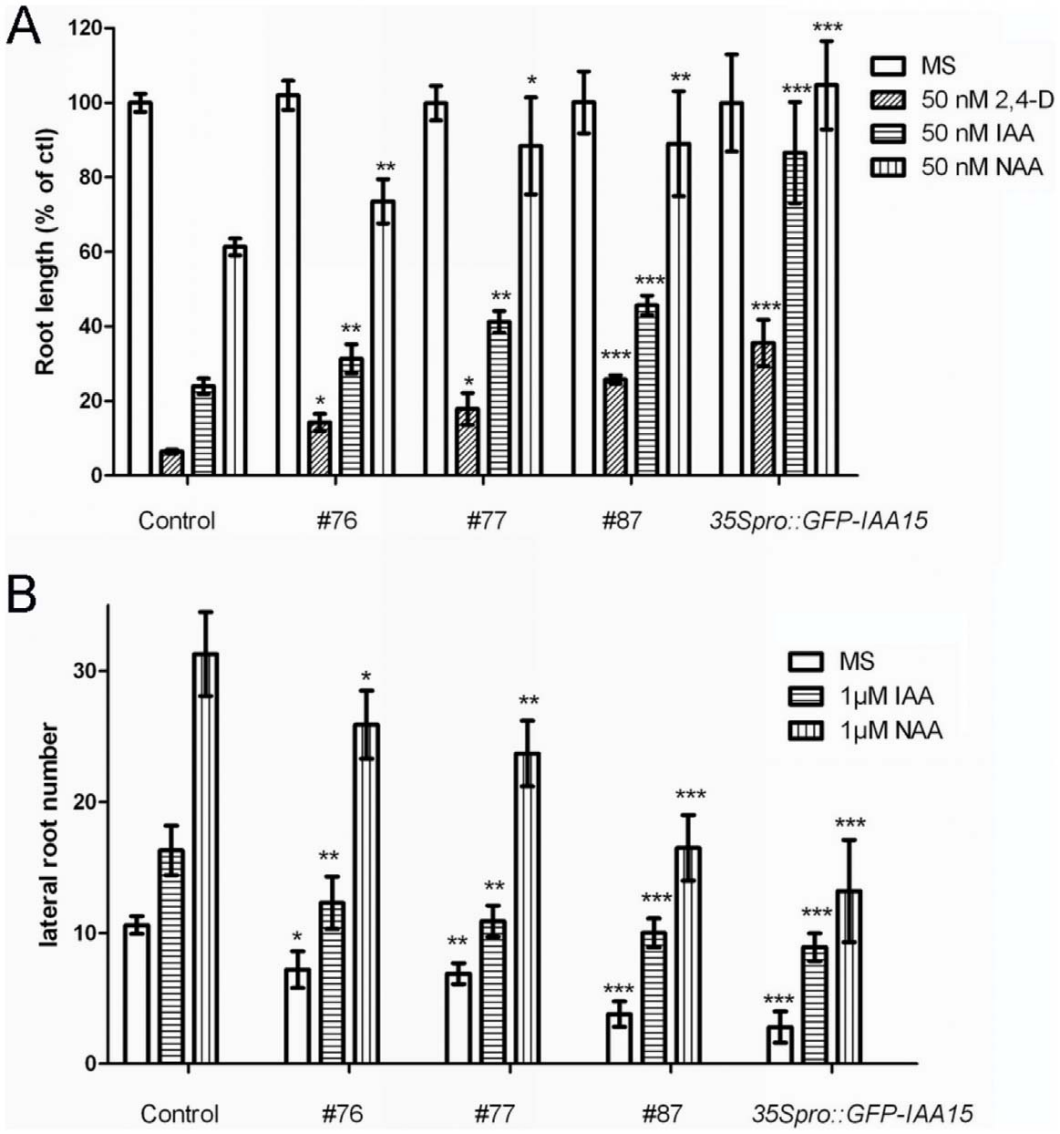
Auxin sensitivity of Col-0 and *IAA15* overexpression lines. Five-day-old control (Col-0) and *IAA15* overexpression lines grown on MS medium were transferred to media containing auxin for an additional 4 days. (A) Root elongation of seedlings on 50 nM 2,4-D or IAA or NAA medium for 4 d. Data are represented as growth of primary roots relative to growth on auxin-free (MS) medium. Error bars represent the SD. A statistical analysis was performed using Student’s *t* test, with significant differences indicated relative to control (*P<0.05, **P<0.01, ***P<0.001). (B) Numbers of emerged lateral roots after 4 d of treatment. Statistical analysis was performed using Student’s *t* test. Asterisks indicate significant differences between control and transgenic lines within treatment (*P<0.05, **P<0.01, ***P<0.001).

Phylogenetic analysis demonstrates that most *Aux/IAAs* subfamilies contain several members within each of their respective subfamilies. *IAA15*(At1g80390) does not follow this pattern, as it is the only member of its subfamily [67]. To elucidate whether the expression levels of *Aux/IAAs* can modulate auxin homeostasis, full-length cDNA of *IAA15* was amplified and overexpressed. Ten independent T1 lines (harboring the *35Spro::IAA15* transgene) with higher *IAA15* expression levels were chosen for further analysis and similar results were obtained for all of the transgenic lines (Fig. S1). We first determined if auxin responses were impaired in the overexpression lines. Primary root elongation and lateral root formation in response to exogenous auxin was examined. Five-day-old wild-type and overexpression lines grown on auxin-free medium were transferred to auxin-free or auxin-containing medium and incubated for an additional four days. The transgenic lines showed reduced auxin sensitivity in both primary root elongation and lateral root formation (Fig. 1), suggesting that they may have an impaired response to auxin. The data displayed below was obtained from line #87 (referred to as *35S_pro_::IAA15*), and used as a representative of our results.

**Figure 2:**
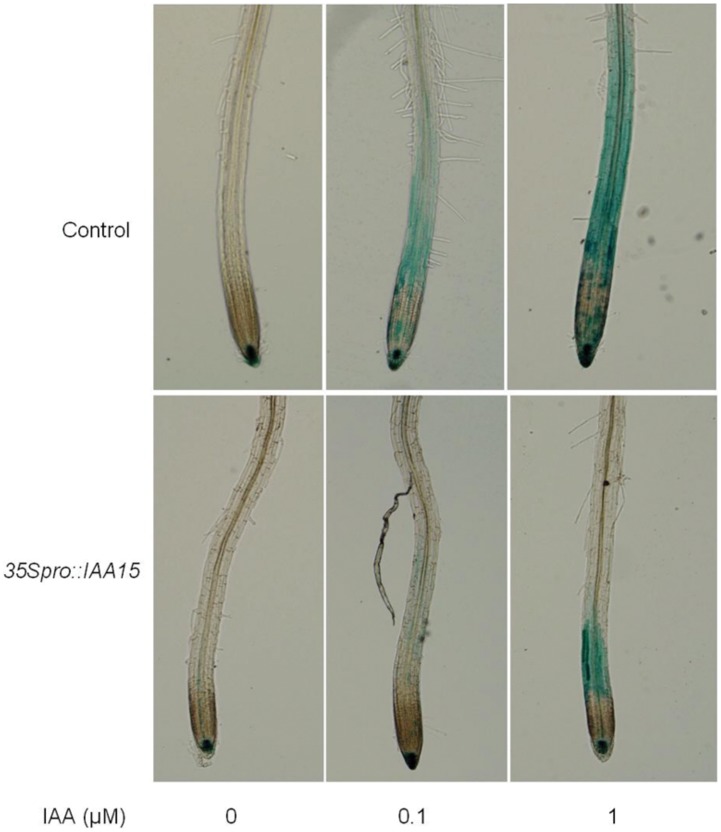
*DR5::GUS* staining in control and *35S_pro_::IAA15* backgrounds in response to IAA treatment. The *DR5::GUS* line was crossed with the *35S_pro_::IAA15* transgenic line and the progeny harboring the *35S_pro_::IAA15* construct, and homozygous for GUS or GFP/YFP/CFP (see below) were collected. Each seedling was genotyped by using PCR primers that detected the *35S_pro_::IAA15* transgene. Aerial tissue was used as the genome template for PCR, except when quantitative real-time PCR and IAA content measurements were performed. Four-day-old seedlings grown on MS medium were transferred to media containing indicated concentrations of IAA for 4 hours and subjected to the staining for 1 hour.

Auxin response was visualized with the marker line *DR5::GUS* [54]. *DR5::GUS* was crossed with the *35S_pro_::IAA15* line and the progeny were further analyzed. While the intensity of GUS staining in the control showed a strong increase in response to exogenous IAA, the *35S_pro_::IAA15* line exhibited dramatically impaired auxin induction of *DR5::GUS* activity (Fig. 2). This is consistent with the reduced auxin sensitivity in both primary root elongation and lateral root formation that was found in the overexpression lines (Fig. 1).

**Figure 3:**
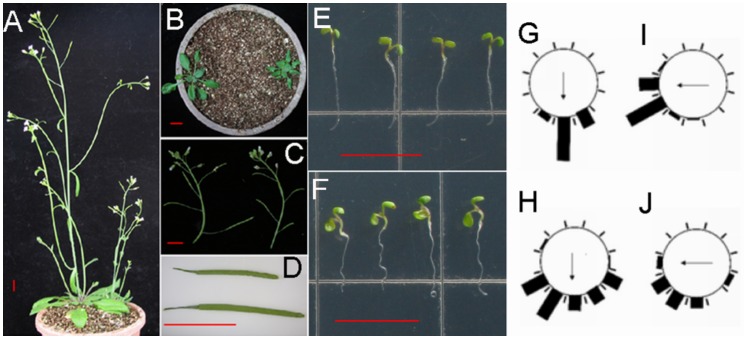
Auxin-related phenotypes of *35S_pro_::IAA15* **transgenic lines.** (A) Reduced apical dominance in five-week-old *35S_pro_::IAA15* transgenic lines (right) compared with that of the wild-type (left). (B) Small and curled leaves of three-week-old *35S_pro_::IAA15* transgenic lines (right) compared with leaves of wild-type (left). (C) Inflorescence with short siliques of five-week-old *35S_pro_::IAA15* transgenic lines (right) in comparison with that of the wild-type (left). (D) A crinkled silique of *35S_pro_::IAA15* transgenic lines (top) compared with a silique of the wild-type (bottom). Root curvatures of four-day-old wild-type (G) and *35S_pro_::IAA15* transgenic lines (H) were assigned to one of the twelve 30° sectors and each bar represents the percentage of seedlings showing the same direction (n>50). After a 90° rotation and growth for another twelve hours, root curvatures of wild-type (I) and *35S_pro_::IAA15*5 transgenic lines (J) were assayed in the same way. Representative seedlings of (I) and (J) are shown in (E) and (F) respectively. Arrows indicate the direction of gravity. Bars: 1 cm.

When *IAA15* was over expressed, dramatic changes in phenotypes were observed in the transgenic plants. Most of these changes were similar to the effects of overexpression of non-canonical Aux/IAAs [60]. In the aerial parts, the *35S_pro_::IAA15* transgenic lines produced short inflorescences with reduced apical dominance (Fig. 3A), small curled leaves (Fig. 3B), and short crinkled siliques containing fewer seeds (Fig. 3C, and 3D). The roots of the transgenic lines exhibited reduced elongation and agravitropic phenotype (Fig. 3F). All of these phenotypes are associated with auxin-related mutants, especially gain-of-function mutants of *Aux/IAAs* [27], [63], [68], [69]. In addition, *IAA15* may function in a dose-dependent manner as some progeny of the *35S_pro_::IAA15* transgenic lines had more severe defects than their sister plants (Fig. S2).

To further understand the role of *IAA15*, T-DNA insertion lines were obtained. Unfortunately, no significant reduction of *IAA15* was observed in these lines. We then decided to use artificial microRNA (amiRNA) transgenic approach, to knock down the endogenous *IAA15* expression [70]. More than 20 independent transgenic lines were subsequently obtained, from which 8 lines were chosen for further analysis of their *IAA15* mRNA expression (Fig. S3). Line #1 and #55, which had the lowest expression of *IAA15,* were further analyzed. There was no significant change of either primary root length, lateral root numbers or gravity responses in line #1 and #55, suggesting that *IAA15* functions redundantly with other *Aux/IAAs* [67].

#### Agravitropic responses in *35Spro::IAA15* transgenic lines

**Figure 4:**
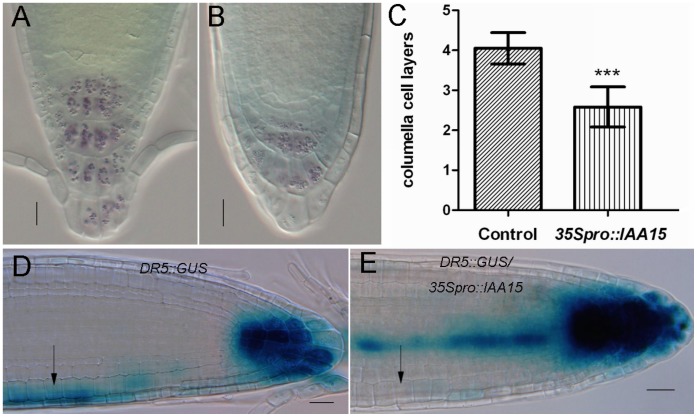
Failure in gravity sensing and auxin response for gravitropism in *35S_pro_::IAA15* transgenic lines. (A–B) Columella cells in the root tip of representative wild-type (A) and *35S_pro_::IAA15* transgenic lines (B) after Lugol’s staining. (C) The number of columella cell layers is 2.58±0.50 in five-day-old seedlings of *35S_pro_::IAA15* transgenic lines (n = 20) in comparison with 4.05±0.39 in that of the wild-type (n = 20), ***P value <0.0001. (D, E) Auxin response is marked by GUS staining in the roots of *DR5::GUS* control (D) and *DR5::GUS/35S_pro_::IAA15* (E) seedlings horizontally placed for six hours and GUS stained for another three hours. The arrow in (D) shows the increase of staining intensity at the bottom of root in *DR5::GUS* control while the arrow in (E) shows the same part of *35S_pro_::IAA15* unchanged in response to gravity. Bars: 20 µm.

Since *35S pro::IAA15* showed agravitropic phenotype (Fig. 3E–3H), further analyses were performed to understand the mechanistic basis of this response. The agravitropic phenotype was more obvious after a 90°rotation was made for another twelve hours (Fig. 3I, and 3J). Starch-rich amyloplasts in columella cells work as statolith to sense gravity and can be stained by Lugol’s solution [12]. Lugol’s staining assay was tested to investigate whether amyloplasts were affected in overexpression lines. The root tip of *35S_pro_::IAA15* transgenic lines had less stained tiers compared with four tiers in the wild-type (Fig. 4A, and 4B). The overall reduction of starch-rich columella cell layers in *35S_pro_::IAA15* transgenic lines (Fig. 4C) suggests that the defect in the gravity response began as early as gravity sensing.

It has been reported that auxin plays a role in gravitropism of *Arabidopsis* roots [32], [71]. We placed *DR5::GUS*, and *DR5::GUS/35S_pro_::IAA15* lines horizontally for six hours, and compared the GUS staining of both. GUS staining expanded to the lower half of the root in *DR5::GUS* lines after six hours (arrow in Fig. 4D). This was not the case for the *DR5::GUS/35S_pro_::IAA15* transgenic line, as the GUS staining remained unchanged after six hours (arrow in Fig. 4E). In summary, these results suggest that the agravitropic phenotype of *35S_pro_::IAA15* transgenic lines results from the impaired auxin response after gravity stimulation. This is likely due to a defect in gravity sensing during gravitropism.

#### Reduced meristem size in *35Spro::IAA15* transgenic lines

**Figure 5:**
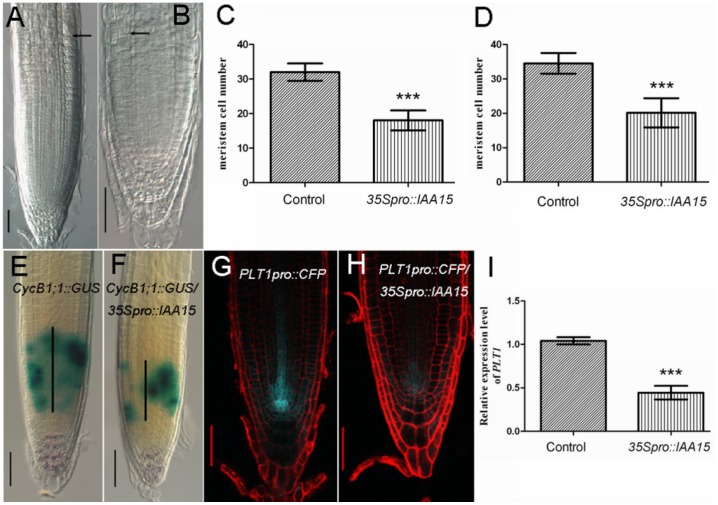
Root meristem size decreases in *35S_pro_::IAA15* transgenic lines. (A, B) A representative seedling with decreased root meristem size in *35S_pro_::IAA15* transgenic line (B) compared with that of the wild-type (A). Root meristem size was defined as the number of cortex cells between the cortex stem cell and the first elongated cortex cell (black arrows). (C, D) In five-day-old roots, meristem cell number is 18.04±2.88 (n = 30) in *35S_pro_::IAA15* transgenic lines contrasted with 32.00±2.51 (n = 30) in wild-type (C), ***P value <0.0001. In seven-day-old roots, meristem cell number is 20.14±4.24 (n = 30) in *35S_pro_::IAA15* transgenic lines in comparison with 34.53±3.02 (n = 30) in wild-type (D), ***P value <0.0001. (E, F) Double staining of Lugol’s solution and GUS in six-day-old seedlings of *CycB1;1::GUS* control (E) and *CycB1;1::GUS/35S_pro_::IAA15* (F) indicates that the population of mitosis-dividing cells is decreased in the overexpression line. Black vertical lines represent the expression area of *CycB1;1::GUS*. (G, H) Overexpression of *IAA15* leads to the repression of *PLT1* as the fluorescence of *PLT1::CFP* is substantially decreased in *PLT1::CFP/35S_pro_::IAA15* (H) contrasted with that in *PLT1::CFP* control (G). (I) Real time RT-PCR assay of *PLT1* transcripts in roots of five-day-old wild-type and *35S_pro_::IAA15* transgenic lines. Data is presented as a mean ± SD from three independent assays, ***P value <0.0001. Bars: 50 µm.

Besides the agravitropic response, ectopic expression of *IAA15* caused a reduction of root meristem size (Fig. 5A–5D). In agreement with the reduced cell number of the root meristem, the GUS stained domain of *CycB1;1::GUS* was reduced when it was introduced into the *35S_pro_::IAA15* background (Fig. 5E, and 5F, black bar, 148.1±13.53 µm in the control, n = 42; 62.50±4.2 µm in the overexpression lines, n = 40. P = 0.0002), indicating that the population of mitotically-dividing cells had decreased.

Given that *PLTs* play important roles in controlling meristem size [6], [7], [72], expression levels of *PLT1* were also analyzed. *PLT1_pro_::CFP* was crossed with the *35S_pro_::IAA15* transgenic line and the progeny were further examined by confocal microscopy. The CFP fluorescence signal was significantly decreased in *PLT1_pro_::CFP*/*35S_pro_::IAA15* compared with that of the control (Fig. 5G, and 5H). Real time RT-PCR was used to quantify the expression level of *PLT1* in the wild-type and the overexpression lines. In coincidence with the microscopic observations, transcript of *PLT1* was reduced in the transgenic line compared with the wild-type (Fig. 5I). Reduced cell numbers in the meristematic zone led to a reduction in primary root length in the transgenic lines (Fig. S4).

#### Over expression of *IAA15* inhibits differentiation of stem cells

**Figure 6:**
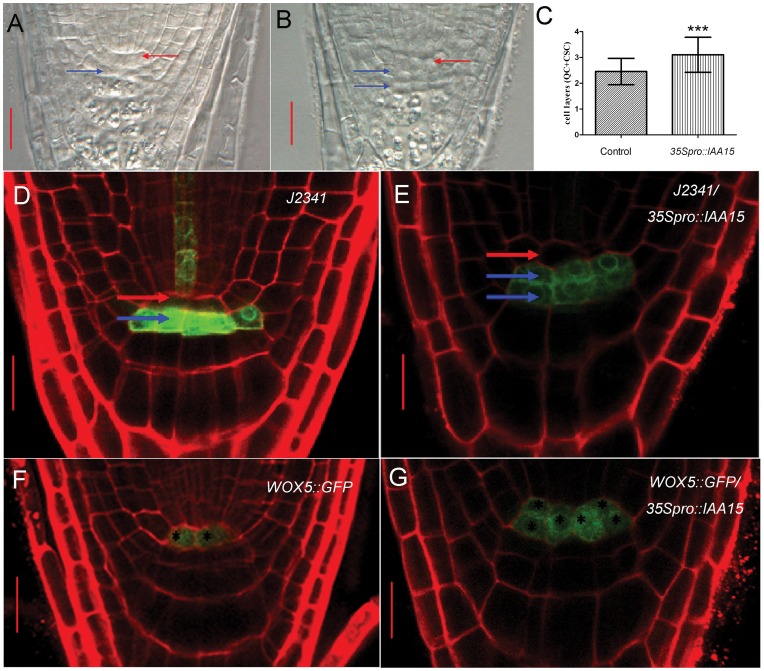
Over expression of *IAA15* inhibits differentiation of stem cells. (A, B) More cell layers are visualized in the root stem cell niche of five-day-old *35S_pro_::IAA15* (B) compared with that of the wild-type (A). (C) The stem cell layers (QC+CSC) of *35S_pro_::IAA15* is 3.10±0.68 (n = 39) compared with 2.45±0.1 (n = 22) in the control. The *t* test, ***P value = 0.0003. (D-G) To further confirm the developmental defects of the root stem cell niche, CSC or QC marker *J2341* and *WOX5::GFP* were introduced into the *35S_pro_::IAA15* background and the progeny were further analyzed. The fluorescence of *J2341* is detected in two cell layers in *J2341/35S_pro_::IAA15* (E) in comparison with one cell layer in *J2341* control (D), which indicates that one more cell layer observed in (B) is undifferentiated CSC. The fluorescence of *WOX5::GFP* is observed in the broader domain including cortex/endodermal initial cells of *WOX5::GFP/35S_pro_::IAA15* (G) in comparison with two QC cells only in *WOX5::GFP* control (F). Stars in (G) and (F) mark the cells expressing *WOX5::GFP*. Red arrows in (A), (B), (D) and (E) indicate the putative QC while blue arrows indicate the putative CSC. Bars: (A, B) 20 µm; (D–G) 10 µm.

The maintenance of the root stem cell niche is vital for root development and the application of auxin promotes the differentiation of CSC [4]. To test whether the auxin response change in the transgenic lines affects the differentiation of stem cells in the root, Lugol’s staining was applied to assay the differentiation of CSC. A decrease in the number of cell layers of columella cells (Fig. 4C) suggests an inhibition of differentiation of CSC in the *35S_pro_::IAA15* transgenic lines. There were more QC and/or CSC cell layers in the *35S_pro_::IAA15* transgenic lines (Fig. 6C). The additional one cell layer visualized in the root stem cell niche could be CSC as marked by the blue arrows in Figure 6. To further confirm these developmental changes, the *35S_pro_::IAA15* transgenic line was crossed with CSC marker line *J2341* (Haseloff enhancer trap GFP line collection) and QC marker *WOX5::GFP.* Confocal microscopy showed that the GFP fluorescence signal of *J2341*/*35S_pro_::IAA15* was detected in two cell layers, in contrast with the control, in which signal was detected in one cell layer (Fig. 6D, and 6E). For QC identity, the GFP fluorescence signal of *WOX5::GFP*/*35S_pro_::IAA15* was visualized. Its expression encompassed a broader domain than that of the control, and included the adjacent cortex/endodermal initial cells. In control roots, *WOX5::GFP* signal was visualized only in two cells (Fig. 6F, and 6G). Taken together, the above results suggest that expanded expression of *WOX5::GFP* promoted the quiescence of the root stem cell niche and repressed the differentiation of CSC in the transgenic lines.

### Perturbation of Auxin Homeostasis in Roots of *35S_pro_::IAA15* Transgenic Lines

**Figure 7:**
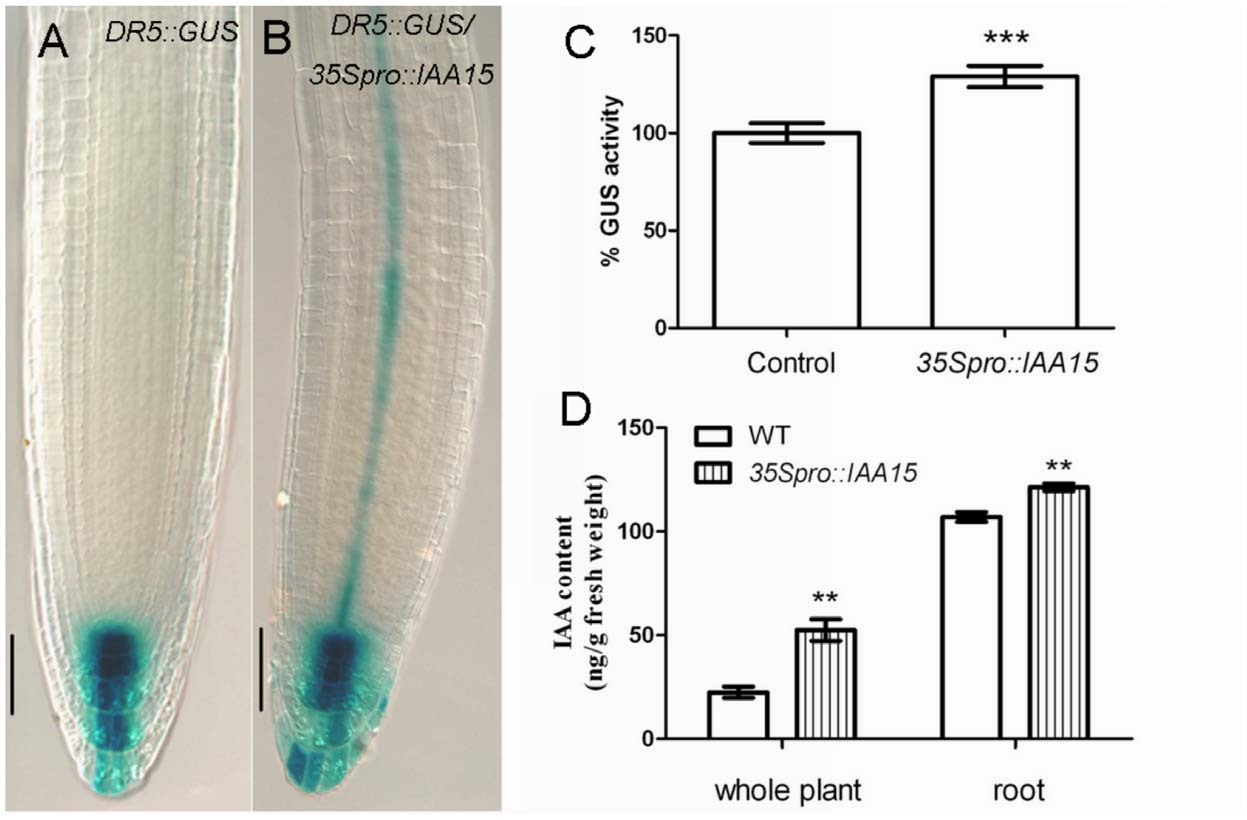
*DR5::GUS* expression and auxin content in *35S_pro_::IAA15* transgenic lines. GUS staining of primary roots for 1.5 hours in five-day-old control (A) and *35S_pro_::IAA15* (B). (C) Quantification of *DR5::GUS* activity. Relative GUS activity was calculated by normalizing to the amount of total protein measured by Bradford assay. (D) Free IAA levels (ng/g fresh weight) in seven-day-old wild-type and *35S_pro_::IAA15*. Data is presented as mean ± SD from three independent assays. **P<0.01, ***P<0.001. Bars: 50 µm.

We next examined whether the auxin homeostasis was affected in the *35S_pro_::IAA15* transgenic lines. It is interesting that the basal expression of *DR5::GUS* is slightly higher in the *35S_pro_::IAA15* background (Fig. 7A–7C). Furthermore, IAA content measurement showed that overexpression confers elevated levels of IAA. The IAA concentration is 52.50 ng/g fresh weight in *35S_pro_::IAA15* compared with 22.48 ng/g fresh weight in the control. In the roots, it is 121.30 ng/g fresh weight in *35S_pro_::IAA15* compared with 107.00 ng/g fresh weight in the control (Fig. 7D).

**Figure 8:**
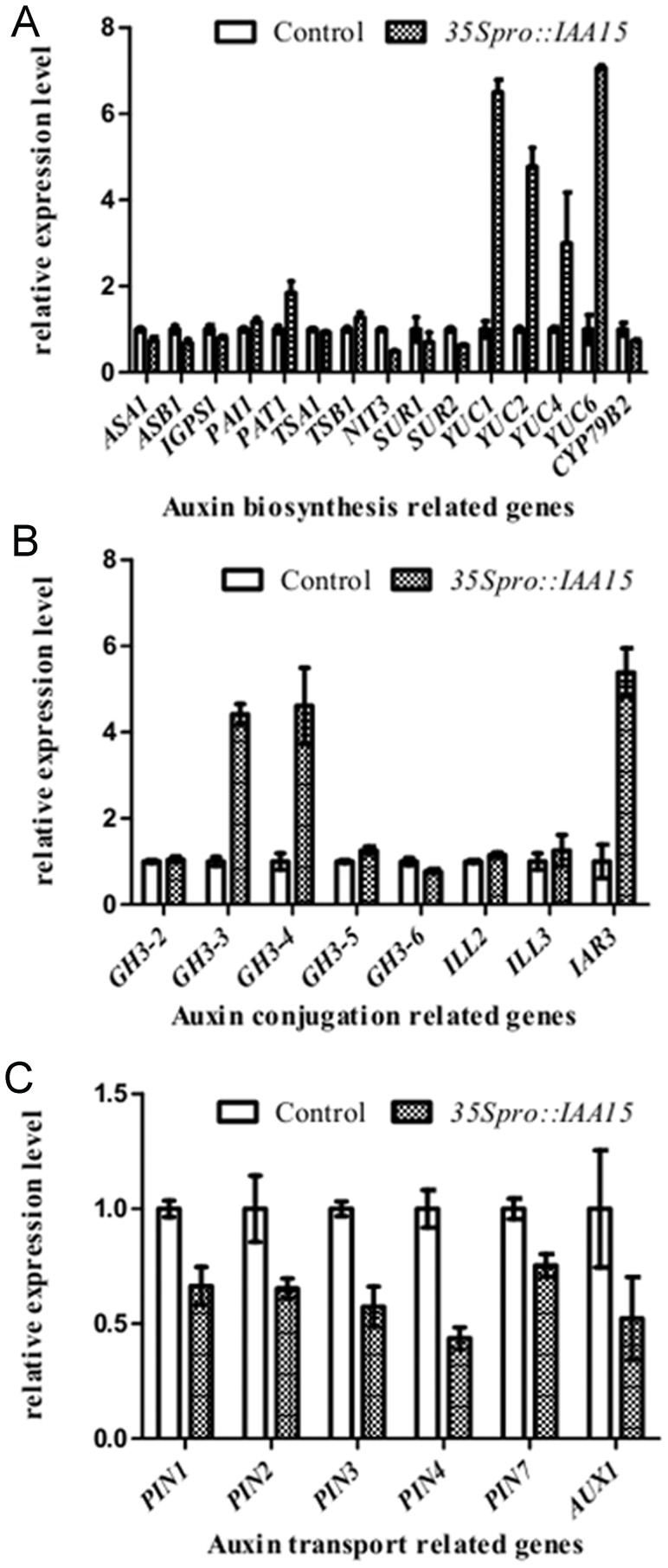
Real time RT-PCR assay of expression of auxin homeostasis related genes. Real time RT-PCR assay of expression of auxin biosynthesis (A), auxin conjugation (B), and auxin transport (C) in roots of five-day-old wild-type and *35S_pro_::IAA15* transgenic lines. Data is presented as a mean ± SD from three independent assays.

As auxin concentration is the integrated output of auxin biosynthesis, PAT, and auxin conjugation, the expression of related genes was monitored by quantitative real-time PCR. Over expression of *IAA15* up regulated the expression of auxin biosynthetic genes *YUC1*, *YUC2*, *YUC4* and *YUC6*, while it down regulated all the genes that regulate auxin transport (Fig. 8A, and 8C). However, different genes for auxin conjugation behaved differentially. One of the IAA-amino acid conjugate hydrolases *IAA-ALANINE RESISTANT 3 (IAR3)*, which releases free IAA by cleaving IAA-amino conjugates, was upregulated while other hydrolases such as *IAA-LEUCINE RESISTANT (ILR)-LIKE GENE 2 (ILL2),* and *ILL3* remained unchanged (Fig. 8B). It is not surprising that IAA-amino synthase *GH3.3* and *GH3.4*, which belong to primary auxin-responsive GH3 family [73], [74], were also upregulated in transgenic lines (Fig. 8B).

**Figure 9:**
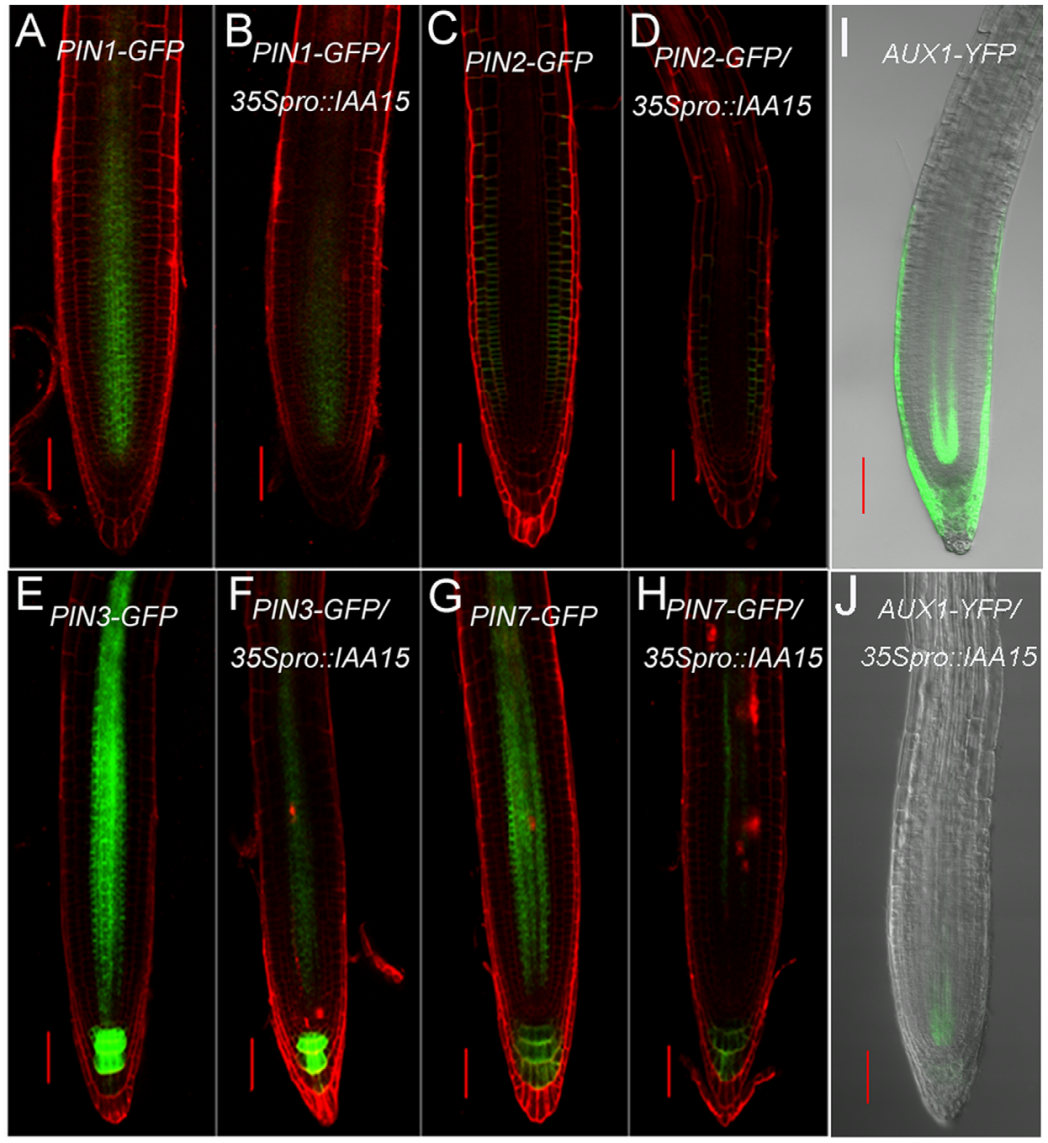
Reduced protein levels of auxin facilitators in *35S_pro_::IAA15* transgenic lines. *35S_pro_::IAA15* transgenic line was crossed with marker lines of auxin facilitators including *PIN1-GFP* (A, B), *PIN2-GFP* (C, D), *PIN3-GFP* (E, F), *PIN7-GFP* (G, H) and *AUX1-YFP* (I, J). Progeny harboring the *35S_pro_::IAA15* construct and homozygous for auxin facilitators were further analyzed by confocal microscopy. In four-day-old roots, the decreased fluorescence was visualized in the *35S_pro_::IAA15* background (B, D, F, H and J) compared with that of the wild-type background (A, C, E, G and I). Bars: 50 µm.

The expanded GUS staining in *DR5::GUS/35S_pro_::IAA15* in the proximal meristem and elongation zone (Fig. 7A), together with reduced transcription levels of *AUX1* and *PINs*, suggests that there may be reduced PAT. To further assay for protein levels of auxin facilitators, *35S_pro_::IAA15* transgenic lines were crossed with marker lines *AUX1_pro_::AUX1-YFP, PIN1_pro_::PIN1-GFP, PIN2_pro_::PIN2-GFP, PIN3_pro_::PIN3-GFP* and *PIN7_pro_::PIN7-GFP* respectively. In agreement with the real-time PCR data, the fluorescence signals of PIN2-GFP, PIN3-GFP, PIN7-GFP and AUX1-YFP were remarkably decreased in *IAA15*-overexpression lines compared with that of the control (Fig. 9C–9J). Slight differences could also be seen for PIN1-GFP fluorescence between control and *IAA15*-overexpression backgrounds (Fig. 9A, and 9B). The reduced PAT was further confirmed by an auxin transport assay. Both acropetal and basipetal IAA transports was reduced in *35Spro::IAA15* transgenic lines (Table 1). Taken together, the above results demonstrate that ectopic expression of *IAA15* influenced PAT, and auxin synthesis and conjugation.

**Table 1:** Reduced basipetal and acropetal auxin transport in *35S_pro_::IAA15* transgenic lines.

IAA transport (fmols)	Control			*35Spro::IAA15*			*P* values^a^	
	−NPA	+NPA	*P* values^b^	−NPA	+NPA	*P* values^b^	−NPA	+NPA
Basipetal	2.53±0.09	1.26±0.10	<0.0001	1.05±0.14	0.87±0.07	<0.0001	<0.0001	0.23
Acropetal	4.75±0.44	1.54±0.13	<0.0001	2.03±0.10	1.34±0.06	0.28	<0.0001	0.01

Basipetal and acropetal movement of IAA in roots was examined by applying [^3^H]-IAA in seven-day-old seedlings, and measuring radioactivity. Data are presented as mean ± SEM from 10 plants. *P* values^a^ (control versus *35Spro::IAA15*) and *P* values^b^ (−NPA versus +NPA) were obtained by Student‘s *t* test.

### The Expression Pattern and Auxin Responsiveness of *IAA15*

**Figure 10:**
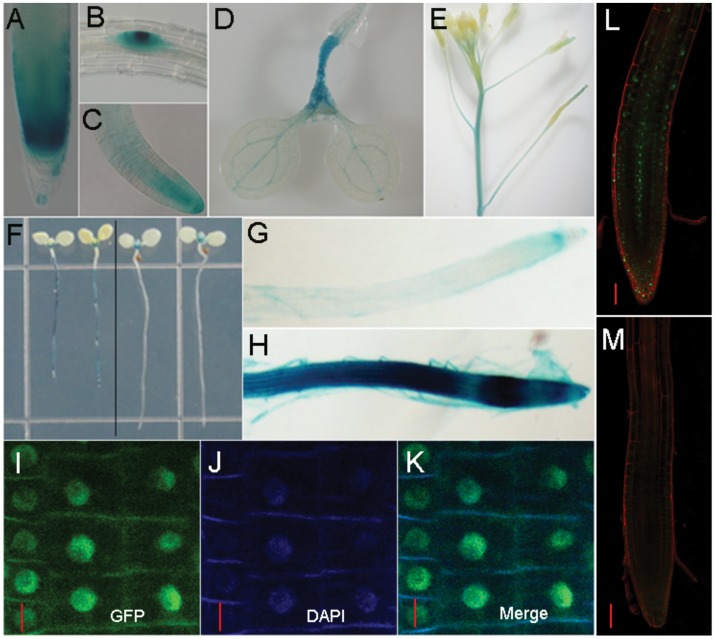
The expression pattern and auxin responsiveness of *IAA15*. (A–E) In *IAA15pro::GUS* transgenic lines, tissue-specific expression of *IAA15* was observed in the primary root (A), lateral root primordia (B), lateral root (C), leaf veins and hypocotyls (D), inflorescence stem and stigma (E). (F–H) *IAA15* could be induced by 10 µM IAA treatment for twelve hours (left part of F), especially in the primary root (H). Mock treatment was carried out using equal volume of ethanol (G and right part of F). (I–K) Subcellular localization of GFP-IAA15 protein in *35S_pro_::GFP-IAA15* transgenic lines. In the roots of four-day-old seedlings, the GFP signal (I) co-localizes with the signal associated with DAPI-stained nuclei (J) in the merged version (K). (L–M) Auxin responsiveness of the translational fusion protein GFP-IAA15. Rapid degradation of GFP-IAA15 in the presence of 100 nM IAA for 10 minutes (M). Mock treatment was carried out using equal volume of ethanol (L). Bars: I-K,10 µm, L-M, 50 µm.

The expression pattern of IAA15 was illucidated through the use of an *IAA15pro::GUS* line. We obtained the β-glucuronidase (GUS) as a reporter gene, and placed it under the control of the *IAA15* promoter. GUS staining was performed as described [75]. All transformants (n = 10) had the same expression pattern with some variation in intensity. *IAA15* was expressed in the columella cells and root meristematic zone, lateral root primordia and lateral root tip (Fig. 10A–10C). In the aboveground regions, *IAA15pro::GUS* lines had GUS staining in the hypocotyls, shoot apical meristem and leaf veins (Fig. 10D). *IAA15* was expressed in the inflorescence stem and stigma during the reproductive phase (Fig. 10E).

Like most of the *Aux/IAAs*, *IAA15* can be induced by auxin. GUS staining drastically permeated the entire root with increased intensity after application of auxin (Fig. 10F–10H). However, there was no difference in the aerial parts suggesting that the induction of *IAA15* by auxin treatment was restricted to the roots (Fig. 10F).

At the subcellular level, the translational fusion protein GFP-IAA15 shared the same location as the cell nucleus, which was stained by DAPI (Fig. 10I–10K). To check whether the GFP-IAA15 abundance is regulated by auxin, *35Spro::GFP-IAA15* seedlings were treated by auxin and analyzed by confocal microscopy. IAA15 was still sensitive to auxin at the protein level, as 100 nM IAA was sufficient for the degradation of GFP-IAA fusion within 10 minutes (Fig. 10L, and 10M).

## Discussion

Previous studies of *Aux/IAAs* focused on the point mutations within conserved domain II, which is required for its rapid proteolysis. *Aux/IAAs* can be targeted by other signals at the transcriptional level. For example, *ARABIDOPSIS RESPONSE REGULATOR 1 (ARR1)* functions downstream of cytokinin in the root transition zone and binds directly to the promoter region of *SHY2/IAA3* to activate its transcription [76]. Elevated levels of *SHY2* can repress *PIN1*, *PIN3,* and *PIN7*, and restrict the effect of auxin outside of the meristematic zone. Besides, *IAA5* and *IAA19* can also be induced by brassinolide [77]. Recently, it was reported that gibberellins negatively regulated the gravitropic reorientation of hypocotyls by transcriptional activation of *IAA5*, *IAA6,* and *MSG2/IAA19,* through degradation of the DELLA proteins [78]. The above evidence suggests that *Aux/IAAs* might mediate the crosstalk between auxin and other signals at the transcriptional level. In this study, we have demonstrated that *IAA15*, an auxin signaling repressor belonging to the Aux/IAAs family, can disturb auxin homeostasis when it is ectopically expressed. Such a high level of transgene expression in *35Spro::IAA15* is not naturally occurring. Nevertheless, the finding in this work may be biologically significant because endogenous signals or environmental cues often modulate auxin signaling by manipulating the expression of more than one *Aux/IAA* [77], [78], [79]. Combinations of these expression changes may be enough to influence auxin homeostasis.

Besides *IAA15*, there exist only a few examples that overexpression of wild-type *Aux/IAAs* can cause noticeable phenotypic changes [65], [66], [80]. The reason why elevated levels of some *Aux/IAAs* do not cause a phenotype could be due to the relatively short-lived nature of these proteins [62]. It is interesting that the GFP-IAA15 fusion was detectable (Fig 10I), suggesting that IAA15 may be a poor substrate for the TIR1/AFBs signaling pathway. However, GFP-IAA15 was still sensitive to auxin as the GFP signal decreased rapidly by IAA treatment (Fig 10L, and 10M).

Aux/IAAs and ARFs are encoded by relatively large gene families with diverse tissue specificities, resulting in a huge complexity in specificity regulation of auxin responses. It is highly likely that the intricate web of protein-protein interactions that mediates auxin responses become disrupted when *IAA15* is ectopically expressed. We can not exclude the possibility that the pleiotropic phenotypes of the *35Spro::IAA15* transgenic lines, were caused indirectly as a result of such a disruption.

Developmental outputs are dependent on both the concentration and gradient of auxin. Auxin can either stimulate or repress root meristem size depending on the concentration and the type of auxin used [81], [82]. On the other hand, some mutants defective in the activity or polar localization of PIN auxin efflux facilitators are dramatically reduced in the root meristem size, suggesting PAT is required for root meristem cell division [83], [84]. With higher endogenous auxin concentration (Fig. 7D) and reduced PAT (Table 1), the *35S_pro_::IAA15* transgenic lines showed reduced expression of *PLT1* as well as a small meristem (Fig. 5). Additionally, the expansion of *WOX5::GFP* signal and reduced differentiation of CSC in *35S_pro_::IAA15* transgenic lines (Fig. 6), indicated that excess auxin failed to promote the differentiation of CSC in the transgenic lines. It is more likely that the increased auxin, is the result of feedback regulation as a result of impaired auxin signaling. In the low-auxin phenotype of the *35S_pro_::IAA15* transgenic lines, an increase in auxin can not overcome the repressive effect of constitutively expressed *IAA15*.

There are various feedback loops between auxin itself and the expression of its transport facilitators [85], [86]. The Aux/IAAs-ARFs signaling pathways are required for the induction of *PIN* genes by auxin treatment. When Aux/IAAs signaling is disrupted, as was seen in the *axr3* or *slr-1* transgenic lines, PIN gene upregulation becomes inhibited in the presence of auxin [87]. In this paper, overexpression of wild-type *IAA15* has a similar repressive response. However, the effect is more severe due to a decrease in the expression of auxin facilitators in the overexpression lines (Fig. 8C, and Fig. 9) compared with nearly normal expression in *slr-1* [87]. Despite the strong reduction of auxin carriers at the protein levels (Fig. 9), overexpression of *IAA15* had only modest effects on them at the transcriptional level (Fig. 8C). This suggests that IAA15 negatively regulates the abundance of auxin carriers in the *35Spro::IAA15* transgenic lines primarily through a post-transcriptional mechanism [88], [89].

## Materials and Methods

### Plant Materials and Growth Conditions

*Arabidopsis thaliana* plants were grown in growth chambers under 16-h light, and 8-h dark condition at 23°C and 120 to 150 µE m^−2 ^s^−1^ illumination. For vertical growth experiments, seeds were surface sterilized, placed in 4°C for 4 days and then transferred to Petri dishes containing half-strength Murashige and Skoog medium (supplemented with 1% agar and 1% sucrose, at pH 5.8) at 23°C and 100 µE m^−2 ^s^−1^ illumination under 16-h light, and 8-h dark condition. Seedlings were analyzed after 3 to 7 days of germination.

Transgenic marker lines used in this paper were previously described in *cycB1;1::GUS* [90]; *DR5::GUS* [54]; *PIN1_pro_::PIN1-GFP* [*91*]; *PIN2_pro_::PIN2-GFP* [83]; *PIN3_pro_::PIN3-GFP* [83]; *PIN7_pro_::PIN7-GFP* [83]; *AUX1_pro_::AUX1-YFP* [34]; *PLT1_pro_::CFP* [6]; *J2341* (http://www.plantsci.cam.ac.uk/Haseloff/) and *WOX5_pro_::GFP* [83].

### Plasmid Construction and Plant Transformation

For *35S_pro_::IAA15*, the coding sequence of *IAA15* was amplified by PCR from cDNA of five-day-old wild-type (Col-0) seedlings and cloned into KpnI and BamHI sites of pCAMBIA1301S [92]. For *35S_pro_::GFP-IAA15* translational fusion, the GFP-IAA15 fragment was obtained by overlapping PCR using GFP and *IAA15* cDNA as templates and cloned into the KpnI and BamHI sites of pCAMBIA1301S. For *amiRNA-IAA15*, amiRNAs were designed to knock down IAA15 using the amiRNA designer interface WMD2 (http://wmd2.weigelworld.org). The amiRNA targeting the “CATAGCAATCGTACATCCCAA” sequence located in the second exon of *IAA15* was chosen and constructed by overlapping PCR using a template plasmid (pRS300) and subsequently cloned into pCAMBIA1301S under the control of a 35S promoter. For *IAA15_pro_::GUS*, a 2.4kb genomic region upstream of the ATG start codon was chosen and cloned into SalI and SmaI sites of pBI101.2 as the promoter for GUS [93]. All primers used for plasmid construction are listed in Table S1. All constructs were confirmed by sequencing and transformed into *Arabidopsis thaliana* (Col-0) by *Agrobacterium tumefaciens* strain GV3101 as previously described [94].

### IAA Concentration Measurement

For IAA quantification analysis, 0.5 g fresh weight of seven-day-old seedlings was immediately frozen in liquid nitrogen. The extraction and purification of endogenous IAA was performed as previously described [95]. The purified samples were methylated by a stream of diazomethane gas, resuspended in 100 µL of ethyl acetate, and analysed by gas chromatography-mass spectrometry-selected ion monitoring (GC-SIM-MS). A Shimadzu GCMS-QP2010 Plus equipped with a HP-5ms column (Agilent, USA) was used to determine the level of IAA. The chromatographic parameters were set as follows: injection temperature at 28°C and initial oven temperature 70°C for 1 min followed by a temperature program of 150°C to 240°C. The standard IAA and D_2_-IAA were purchased from Sigma-Aldrich (MO, USA). The monitored ions were *m/z* 130 and 132 (quinolinium ions from native IAA and D_2_-IAA internal standard respectively), *m/z* 77, 189, and 191 (molecular ion and m^+^+6).

### RNA Isolation and Real Time RT-PCR Assay

For whole seedlings or rosette leaves, RNA extraction was performed as previously described [96]. For roots, RNA extraction was performed using PureLink™ Plant RNA Reagent (Invitrogen) according to the instruction manual. All RNA samples were treated by RQ1 RNase-free DNase I (Promega) to remove DNA contamination and reverse transcription was carried out using ReverTra Ace® (TOYOBO).

Real time RT-PCR assay was performed using CFX96™ Real-Time PCR Detection System (Bio-RAD). PP2A subunit *PDF2* (At1g13320) was chosen as the reference gene by geNorm software [97], [98]. PCR was performed as follows: 3 min at 95°C, followed by 40 cycles of denaturation for 15s at 95°C, annealing for 20 s at 56°C, and extension for 20 s at 72°C. Primers used for the quantitative assay were described previously [99], [100], [101], [102], [103] or displayed in Table S1.

### Microscopic Analysis

For phenotypic observation of root or GUS staining, seedlings were cleared and mounted with clearing solution (8 g of chloral hydrate, 2 mL of water, and 1 mL of glycerol) on glass slides. They were examined under Differential Interference Contrast microscopy (DIC) Olympus BX60 and photographed by Charge Coupled Device (CCD) Olympus dp72.

For starch staining, primary roots were immersed in 20% (v/v) Lugol’s solution (Fluka) for 5 minutes in the dark, and followed three times by washing with ddH_2_O. Prepared roots were cleared and mounted with clearing solution for microscopic observation under DIC.

Confocal microscopy was performed using Olympus FluoView 1000-confocal laser scanning microscope according to the manufacturer’s instructions. GFP and CFP lines were mounted with 20 µg mL^−1^ Propidium iodide (PI) while YFP lines were mounted with ddH_2_O. For 4′, 6-diamidino-2-phenylindole (DAPI) staining, seedlings were immersed in 5 µg mL^−1^ DAPI for 10 minutes and mounted with Antifade Mounting Medium (0.5 M sodium carbonate-bicarbonate buffer, pH 9.5 diluted with equal volume of glycerol) after washing three times with ddH_2_O.

### Auxin Transport Assays

Basipetal and acropetal auxin transport measurements were performed in Col-0 wild-type and *35S_pro_::IAA15* as previously described [104]. Seven-day-old seedlings were moved and aligned on a fresh plate. For basipetal auxin transport assays, an agar line containing 100 nM [^3^H]-IAA (Amersham) was applied to aligned root tips. After vertically placed in the dark for 5 hours, the first 2-mm of the root tip touching the radioactive agar were discarded and 5-mm sections from the root tip were cut and assayed for radioactivity by scintillation counting. For acropetal auxin transport assays, an agar line containing 100 nM [^3^H]-IAA (Amersham) was applied to the region just below shoot-root junction. Plates were inverted upside down and vertically incubated in the dark for 18 h. Subsequently, agar lines were removed and 5-mm sections from the root tip were cut and assayed.

## Supporting Information

Figure S1 **Characterization of** ***IAA15*** **overexpression lines.** (A) Quantitative real-time PCR of *IAA15* transcripts in different transgenic lines. RNA was extracted from rosette leaves of 4-week old plants and reverse transcribed to cDNA (see Materials and Methods for details) for real-time PCR analysis. Primary root length (B) and meristem cell number (C) in seven-day-old seedlings of different transgenic lines. (D) Lateral root number in ten-day-old seedlings of different transgenic lines. Data is presented as mean ± SD from three independent assays. Asterisks indicate significant differences between control and transgenic lines (*P<0.05, **P<0.01, ***P<0.001).
(TIF)

Figure S2 ***IAA15*** **may function in a dose-dependent manner.** (A) Five-week-old seedlings of wild-type and two progeny of the *35S_pro_::IAA15*. Progeny #2 had more severe defects than progeny #1. (B) Quantitative real-time PCR of *IAA15* transcripts in progeny #1 and progeny #2. RNA was extracted from rosette leaves and reverse transcribed to cDNA for real-time PCR analysis. Data is presented as mean ± SD from three independent assays. (C) Ten-week-old plant of progeny #2 was sterile and generated empty siliques. Some progeny of the *35Spro::GFP-IAA15* had the same phenotype. (D-G) Other independent transgenic lines with higher *IAA15* transcripts than the *35Spro::GFP-IAA15* (>2 fold) were sterile in the T1 generation.
(TIF)

Figure S3 **Characterization of** ***amiR-IAA15*** **lines.** (A) Quantitative real-time PCR of *IAA15* transcripts in different *amiR-IAA15* lines. Primary root length (B) and lateral root number (C) in ten-day-old seedlings of control and two *amiR-IAA15* lines. (D) Gravity response of control and two *amiR-IAA15* lines. Seedlings were vertically grown for 4 days and reoriented by 90° for 12 hours. Data is presented as mean ± SD from three independent assays.
(TIF)

Figure S4 **Reduced primary root length of the** ***35S_pro_::IAA15*** **.** Primary root length of wild-type (open cycles) and *35S_pro_::IAA15* (filled cycles) at different days after germination. Error bars represent SD from three different experiments.
(TIF)

Table S1 **Sequences of primers.**
(DOC)
