# Supplementary figures and images for: Perturbation of Auxin Homeostasis by Overexpression of Wild-Type IAA15 Results in Impaired Stem Cell Differentiation and Gravitropism in Roots
Source: PLoS One. 2013 Mar 5;8(3):e58103. doi: 10.1371/journal.pone.0058103 (PMC3589423; doi:10.1371/journal.pone.0058103)

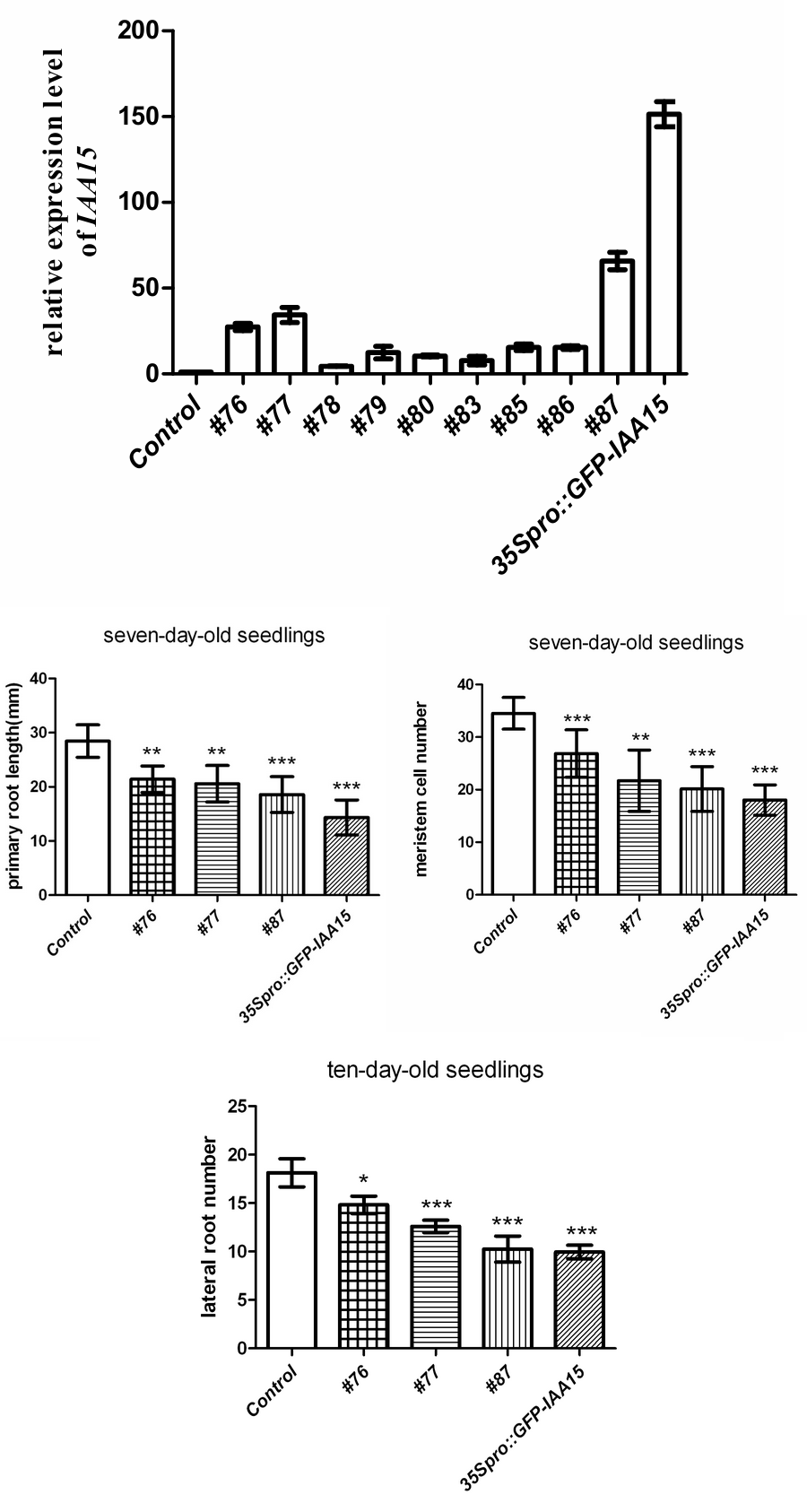

Supplement: Figure S1 — Characterization of IAA15 overexpression lines. (A) Quantitative real-time PCR of IAA15 transcripts in different transgenic lines. RNA was extracted from rosette leaves of 4-week old plants and reverse transcribed to cDNA (see Materials and Methods for details) for real-time PCR analysis. Primary root length (B) and meristem cell number (C) in seven-day-old seedlings of different transgenic lines. (D) Lateral root number in ten-day-old seedlings of different transgenic lines. Data is presented as mean ± SD from three independent assays. Asterisks indicate significant differences between control and transgenic lines (*P<0.05, **P<0.01, ***P<0.001). (TIF) [file pone.0058103.s001.tif]

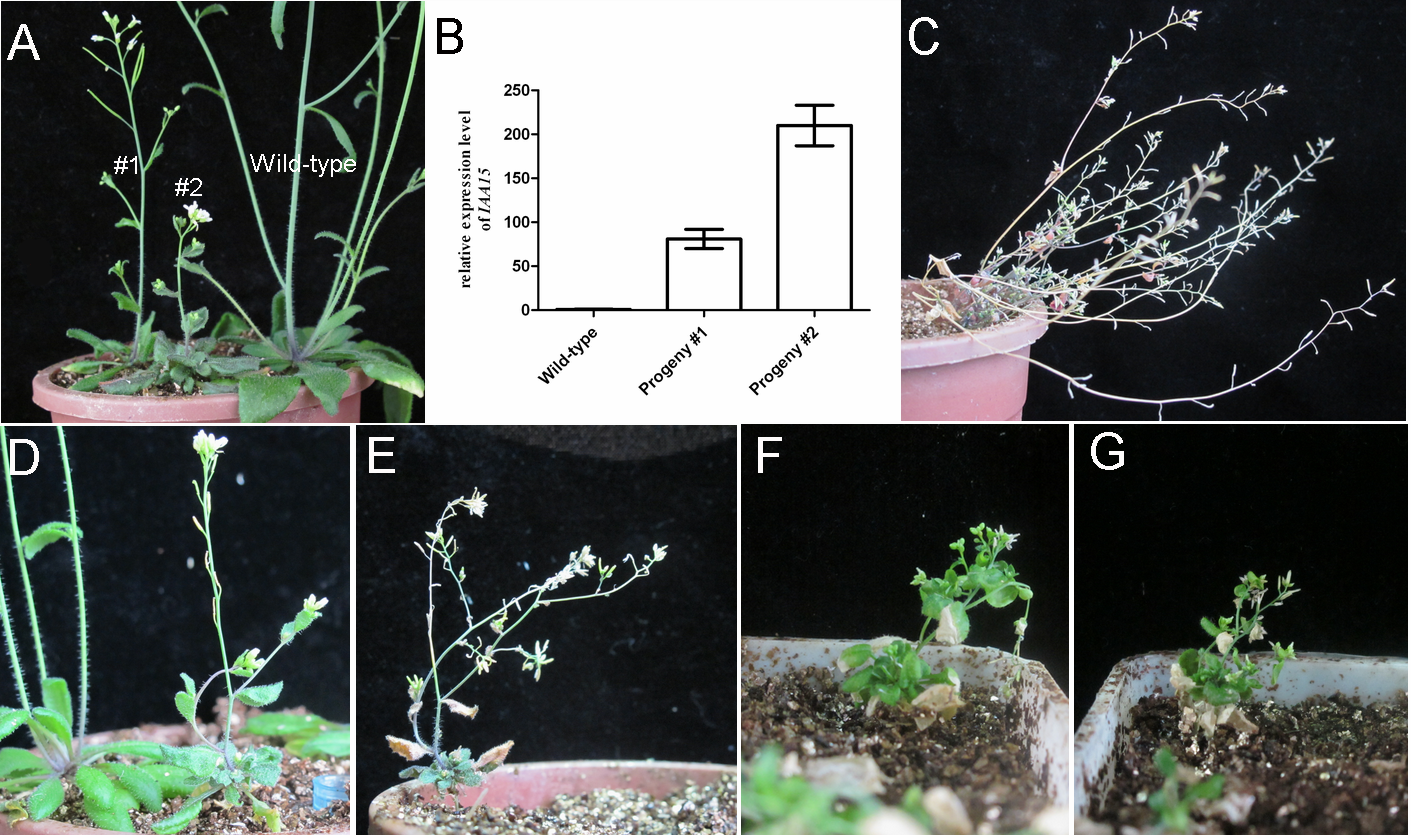

Supplement: Figure S2 — IAA15 may function in a dose-dependent manner. (A) Five-week-old seedlings of wild-type and two progeny of the 35Spro::IAA15. Progeny #2 had more severe defects than progeny #1. (B) Quantitative real-time PCR of IAA15 transcripts in progeny #1 and progeny #2. RNA was extracted from rosette leaves and reverse transcribed to cDNA for real-time PCR analysis. Data is presented as mean ± SD from three independent assays. (C) Ten-week-old plant of progeny #2 was sterile and generated empty siliques. Some progeny of the 35Spro::GFP-IAA15 had the same phenotype. (D-G) Other independent transgenic lines with higher IAA15 transcripts than the 35Spro::GFP-IAA15 (>2 fold) were sterile in the T1 generation. (TIF) [file pone.0058103.s002.tif]

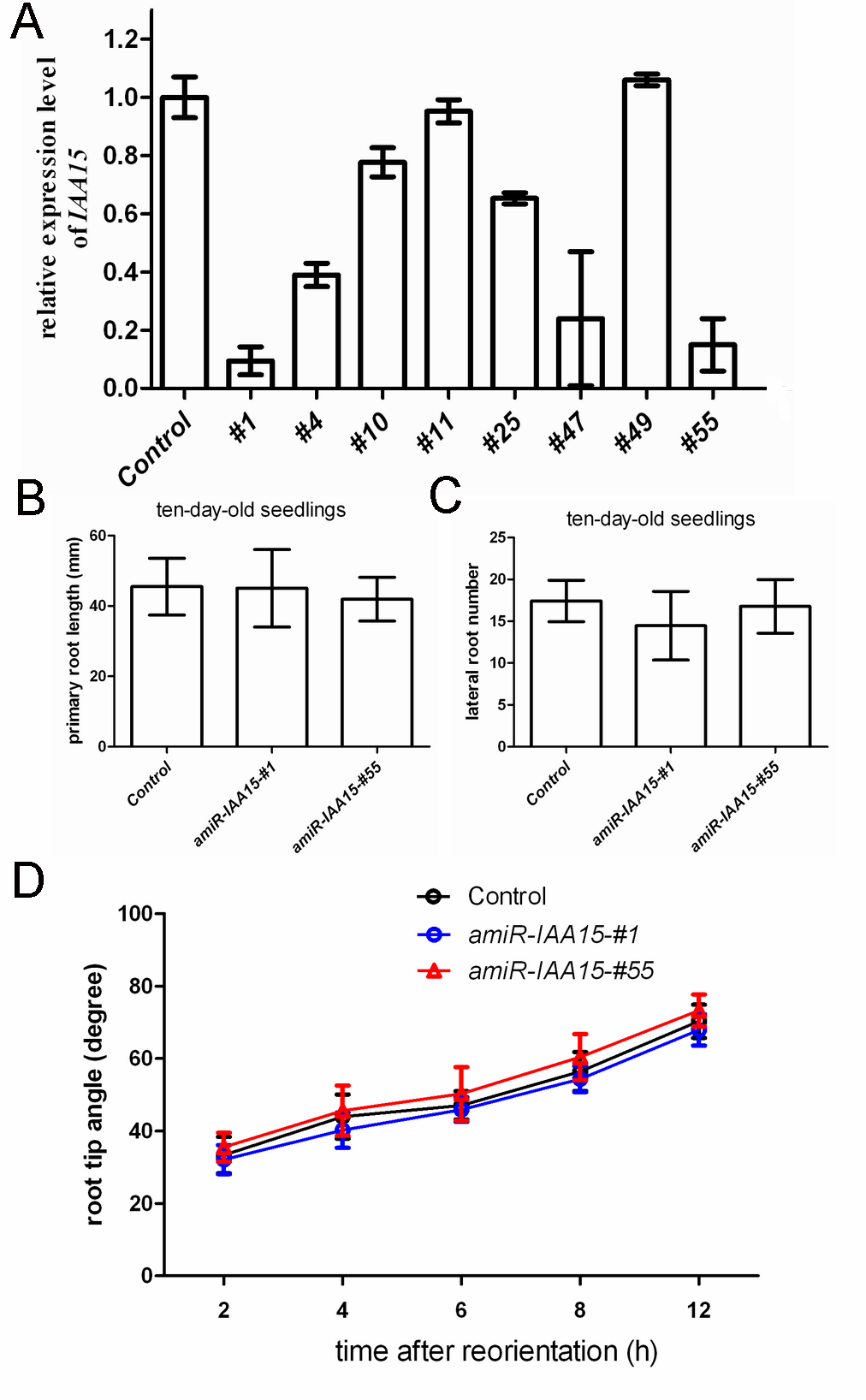

Supplement: Figure S3 — Characterization of amiR-IAA15 lines. (A) Quantitative real-time PCR of IAA15 transcripts in different amiR-IAA15 lines. Primary root length (B) and lateral root number (C) in ten-day-old seedlings of control and two amiR-IAA15 lines. (D) Gravity response of control and two amiR-IAA15 lines. Seedlings were vertically grown for 4 days and reoriented by 90° for 12 hours. Data is presented as mean ± SD from three independent assays. (TIF) [file pone.0058103.s003.tif]

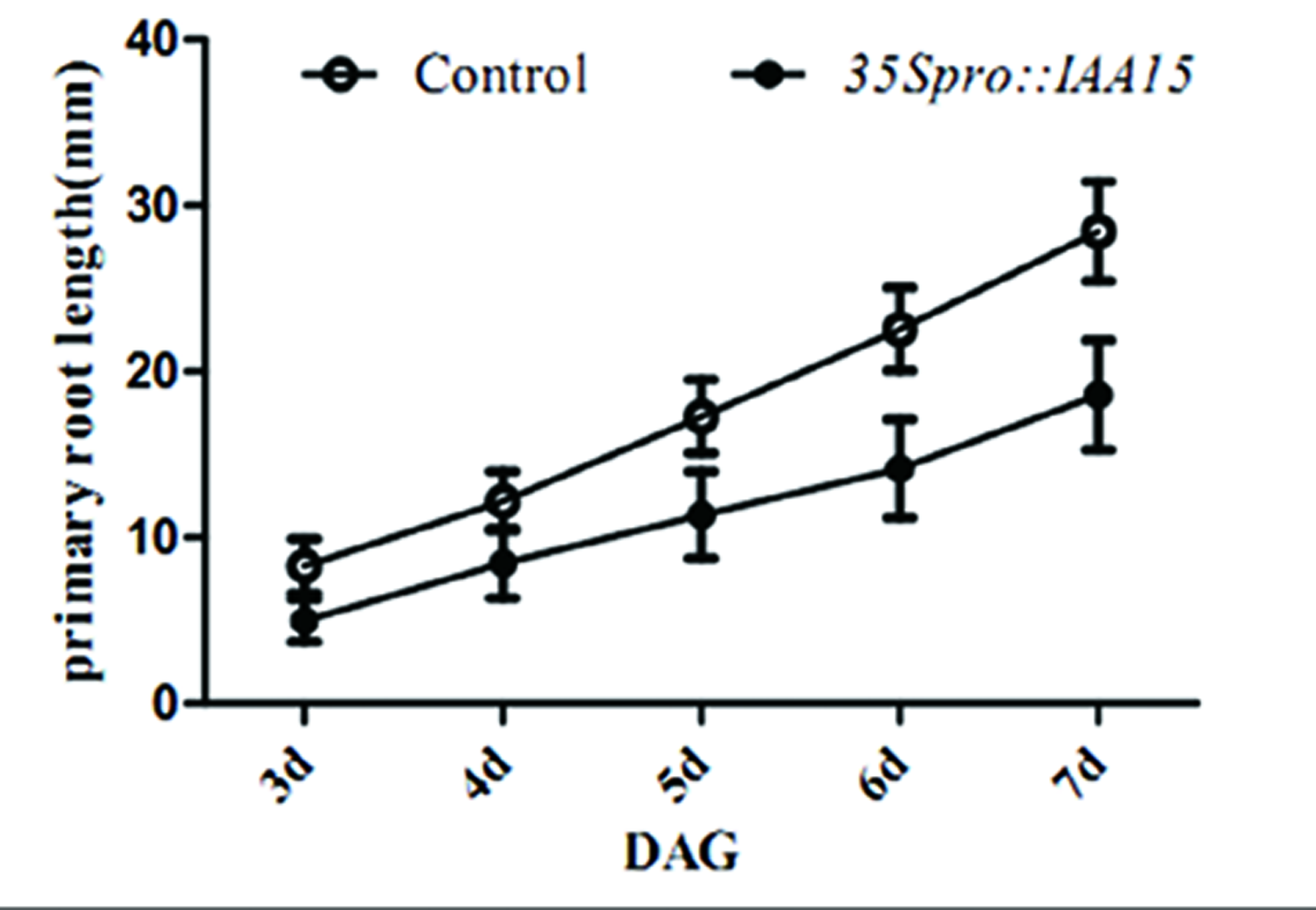

Supplement: Figure S4 — Reduced primary root length of the 35Spro::IAA15 . Primary root length of wild-type (open cycles) and 35Spro::IAA15 (filled cycles) at different days after germination. Error bars represent SD from three different experiments. (TIF) [file pone.0058103.s004.tif]
