# Supplementary material for: Perturbation of Auxin Homeostasis by Overexpression of Wild-Type IAA15 Results in Impaired Stem Cell Differentiation and Gravitropism in Roots
Source: PLoS One. 2013 Mar 5;8(3):e58103. doi: 10.1371/journal.pone.0058103 (PMC3589423; doi:10.1371/journal.pone.0058103)
Supplement: Table S1 — Sequences of primers. (DOC) [file pone.0058103.s005.doc]

**Table S1** Sequences of primers

| Primer name | Primer sequence (5’-3’) |
| --- | --- |
| **For *35S_pro_::IAA15*** | |
| IAA15F(KpnI) | GGGGTACCATGTCACCGGAGGAATACGTTAG |
| IAA15R(BamHI) | CGGGATCCTAATCCAATAGCATCTCCGGTTTTC |
| **For GFP-IAA15 overlapping PCR** | |
| GFP-15F | CGAGCTGTACAAGCTCGAGATGTCACCGGAGGAATACGTTAG |
| GFP-15R | CTAACGTATTCCTCCGGTGACATCTCGAGCTTGTACAGCTCG |
| GFPF(KpnI) | GGGGTACCATGGTGAGCAAGGGCGAGGAG |
| **For *IAA15_pro_::GUS*** | |
| IAA15proF(SalI) | ACGCGTCGACCAGTAAATAGGGAGAAGACTGATTG |
| IAA15proR(SmaI) | TCCCCCGGGGTTAGTTAACAACAAAAGTGCGGAAAG |
| **For real-time PCR** | |
| IAR3F | TTGGCTTCTCCTATTCTTCTACTC |
| IAR3R | ACCGTTTCAGCTCTCTCATTG |
| ILL2F | ATGGCAGTACAGTACCTCAAAG |
| ILL2R | TGAACCAATAACCGATGAACAAC |
| ILL3F | GCTTAGTTAATTGCCTTCACAAG |
| ILL3R | TGAGACAGAGACCCATTACTTAC |
| IAA15F | GAGTGACAGAGTTGGAGAGGAAGG |
| IAA15R | ACAAACATCATCCACGGCACATC |
| **For amiR-IAA15** | |
| ImiR-s | gaTATAGCAATCGTACATCCCCAtctctcttttgtattcc |
| IImiR-a | gaTGGGGATGTACGATTGCTATAtcaaagagaatcaatga |
| IIImiR*s | gaTGAGGATGTACGAATGCTATTtcacaggtcgtgatatg |
| IVmiR*a | gaAATAGCATTCGTACATCCTCAtctacatatatattcct |
